# Lignocellulose binding of a Cel5A-RtCBM11 chimera with enhanced β-glucanase activity monitored by electron paramagnetic resonance

**DOI:** 10.1186/s13068-017-0964-0

**Published:** 2017-11-14

**Authors:** Raquel Fonseca-Maldonado, Luana P. Meleiro, Luís F. S. Mendes, Luana F. Alves, Sibeli Carli, Lucas D. Morero, Luis G. M. Basso, Antonio J. Costa-Filho, Richard J. Ward

**Affiliations:** 1 Departamento de Química, Faculdade de Filosofia Ciências e Letras de Ribeirão Preto, Universidade de São Paulo, Avenida Bandeirantes 3900, Ribeirão Preto, São Paulo Brazil; 2 Departamento de Gestão, Instituto Federal de Educação, Ciência e Tecnologia de São Paulo/IFSP Campus Jacareí, Jacareí, São Paulo Brazil; 3 Departamento de Física, Faculdade de Filosofia Ciências e Letras de Ribeirão Preto, Universidade de São Paulo, Ribeirão Preto, São Paulo Brazil; 4 Departamento de Bioquímica, Faculdade de Medicina de Ribeirão Preto, Universidade de São Paulo, Ribeirão Preto, São Paulo Brazil

**Keywords:** Electron paramagnetic resonance, Site-directed spin labeling, Lignocellulose binding, Enzyme engineering

## Abstract

**Background:**

The Bacillus subtilis endo-β-1,4-glucanase (BsCel5A) hydrolyzes β-1,3-1,4-linked glucan, and the enzyme includes a family 3 carbohydrate-binding module (CBM3) that binds β-1,4-linked glucan.

**Methods:**

Here we investigate the BsCel5A β-1,3-1,4 glucanase activity after exchanging the CBM3 domain for the family 11 CBM from Ruminiclostridium thermocellum celH (RtCBM11) having β-1,3-1,4 glucan affinity.

**Results:**

The BsCel5A-RtCBM11 presents a 50.4% increase in Vmax, a 10% reduction in K0.5, and a 2.1-fold increase in catalytic efficiency. Enzyme mobility and binding to barley β-1,3-1,4 glucan and pre-treated sugarcane bagasse were investigated using Electron Paramagnetic Resonance (EPR) with Site-Directed Spin Labeling (SDSL) of the binding site regions of the CBM3 and RtCBM11 domains in the BsCel5A-CBM3 and BsCel5A-RtCBM11, respectively. Although higher mobility than the RtCBM11 was shown, no interaction of the spin-labeled CBM3 with β-1,3-1,4 glucan was observed. In contrast, a Ka value of 0.22 mg/mL was estimated from titration of the BsCel5A-RtCBM11 with β-1,3-1,4 glucan. Enzyme binding as inferred from altered EPR spectra of the BsCel5A-RtCBM11 was observed only after xylan or lignin extraction from sugarcane bagasse. Binding to xylan- or lignin-free lignocellulose was correlated with a 4.5- to 5-fold increase in total reducing sugar release as compared to the milled intact sugarcane bagasse, suggesting that xylan impedes enzyme access to the β-1,3-1,4 glucan.

**Conclusions:**

These results show that the non-specific binding of the BsCel5A-RtCBM11 to the lignin component of the cell wall is minimal, and represent the first reported use of EPR to directly study the interaction of glycoside hydrolyse enzymes with natural insoluble substrates.

**Electronic supplementary material:**

The online version of this article (10.1186/s13068-017-0964-0) contains supplementary material, which is available to authorized users.

## Background

Cellulose is the major component of the plant cell wall, and is integrated with various other polysaccharides and lignin to form a complex polymer matrix. Cellulose forms linear chains comprising glucose monomers linked by β-1,4-glycosidic bonds [1]. Abundant hydrogen bonding both within and between the cellulose polymers imposes a largely crystalline structure that is recalcitrant to degradation [2]. The enzymatic hydrolysis of cellulose requires a consortium of glycoside hydrolases initiated by the action of endoglucanases (endo-1,4-β-glucosidases: EG; EC 3.2.1.4) that cleave β-1,4-glycosidic bonds in the regions of the cellulose fibers showing low crystallinity, which results in the release of oligosaccharides, cellobiose, and glucose. Subsequently, exoglucanases or cellobiohydrolases (exo-1,4-β-glucosidases; CBH; EC 3.2.1.91) act on the reducing and non-reducing extremities in crystalline regions of the cellulose fibers to release glucose and cellobiose, and finally β-glucosidases hydrolyze short cell oligosaccharides and cellobiose to release glucose [3]. This synergistic hydrolysis of cellulose contributes to carbon recycling in the biosphere [3–5], and has been extensively studied from the viewpoint of biotechnological applications in the biofuels, animal feed, pulp bleaching, wastewater treatment, and paper bioconversion industries [6, 7]. The use of enzymes can reduce energy costs and avoid the use of chemical processes that could ultimately harm the natural environment [8].

Endo- and exoglucanases frequently comprise two or more domains that display multi-functional properties, where a catalytic domain (CD) is responsible for the hydrolytic activity, and non-catalytic domains enhance catalysis or perform structural functions [9]. The non-catalytic modules include the Carbohydrate-Binding Modules (CBMs) [10], which are coupled to the catalytic domains via flexible polypeptide linkers [11]. It is widely recognized that the CBM domains of cellulases improve the efficiency of enzymatic hydrolysis of insoluble substrates [12] as a result of their functions in polysaccharide recognition and binding [13]. The CBMs are currently grouped into 81 families (CAZy database, http://www.cazy.org) that display variable binding specificities against different plant cell wall polysaccharides such as crystalline or amorphous cellulose, β-glucans, xylans, laminarins, mannans, galactans, xyloglucans, arabinans, and others.

Electron Paramagnetic Resonance (EPR) spectroscopy is a well-established technique, which targets defined paramagnetic centers [14] and is not subject to interference from signal scattering resulting from the presence of particulate material [15]. EPR spectroscopy together with site-directed spin labeling (SDSL) has been used to investigate structural and conformational changes of proteins [16–18]. The use of EPR is well suited to study the binding of spin-labeled proteins to lignocellulosic biomass, and in contrast to Nuclear Magnetic Resonance (NMR) and X-ray crystallography, the technique can be applied under conditions found *in natura* [19] or under high solids loading. In SDSL, an extrinsic spin probe is covalently attached to the thiol side chain in a native or site-directed mutated Cys residue [16], and analyses of the EPR spectrum can provide information with respect to protein chain dynamics, solvent accessibility, local polarity, and protein interactions under the chosen experimental conditions [14, 20–24]. Furthermore, when multiple probes are introduced into a protein, EPR-SDSL can be used to measure distances between the spin probes within the polypeptide chain [25, 26].

The thermostable *Bacillus subtilis* cellulase 5A (BsCel5A) is a well-characterized endo-β-1,4-glucanase, and comprises a family 5 glycoside hydrolase (GH5) catalytic domain fused to a family 3 carbohydrate-binding module (CBM3) [27]. The CBM3 family has an extended planar binding surface, and has been proposed to interact mainly with insoluble substrates such as crystalline cellulose [10]. Biochemical characterization of BsCel5A showed a maximum enzymatic activity at pH 6 and 60 °C, and a high thermal stability [27]. Although the BsCel5A hydrolyzes the β-1,4 linkages both in soluble carboxymethylcellulose (CMC) and in crystalline cellulose, the enzyme has a 5-fold higher catalytic efficiency against barley β-glucan and lichenan [27], which are soluble polysaccharides composed of β-1,3-β-1,4 mixed linked glucose monomers [28].

The CelH of *Ruminiclostridium thermocellum* (previously *Clostridium thermocellum*) is a multidomain protein [29], which encodes an N-terminal GH26 β-1,3-1,4-glucanase catalytic domain, a second catalytic GH5 β-1,4-cellulase domain, a family 11 carbohydrate-binding module (RtCBM11), and a dockerin domain at the C-terminus [29, 30]. Since the RtCBM11 domain has high affinity for β-glucan polymers, we surmised that its fusion with the catalytic domain of BsCel5A could increase the β-1,3-1,4-glucanase activity of the enzyme. Here we report the application of EPR-SDSL to investigate the dynamics and binding characteristics of the BsCel5A catalytic domain fused with the carbohydrate-binding modules CBM3 and RtCBM11 in the presence of the natural substrate barley β-glucan and several types of pre-treated sugarcane bagasse.

## Methods

### Cloning, expression, and purification

Expression systems in *E. coli* using the pET28a vector have been previously described for the *Bacillus subtilis* BsCel5A-CBM3 [27] and the RtCBM11 from the *cel*H of *R. thermocellum* [31]. The RtCBM11 nucleotide sequence cloned in the pET28a plasmid was used as template for amplification with oligonucleotides CBM_Bsp_F (containing restriction site BspI) and the T7 terminator sequence (containing restriction site BamHI). The amplified fragment was purified from 1% agarose gels using the GeneJET kit (Fermentas, Vilnius, Lithuania), and digested with restriction enzymes BspI and BamHI. Digestion of the BsCel5A-CBM3_pET28a construct with the same two restriction enzymes liberates the sequence encoding the CBM3 domain, and generates compatible cohesive ends for ligating the digested RtCBM11 PCR fragment. Following ligation, the integrity of the BsCel5A-RtCBM11_pET28a construct was confirmed by automated nucleotide sequencing.

Competent *E. coli* Rosetta BL21 (DE3) cells were transformed with either the BsCel5A-CBM3 or the BsCel5A-RtCBM11. After regeneration, the transformed cells were cultured in 10 mL of selective LB liquid at 37 °C for 16 h. These cultures were used to inoculate 1 L of selective LB liquid medium, and after an appropriate cell density had been reached (OD_600_ = 0.7) protein expression was induced by the addition of 0.5 mM IPTG (isopropyl-β-d-thiogalactopyranoside) and ran for 6 h under the same culture conditions. Subsequently, the cells were harvested by centrifugation, and lysed by sonication in buffer containing 40 mM HEPES, 300 mM NaCl, pH 7.5, and 40 mM imidazole. After centrifugation to clear cell debris, the supernatant was incubated with 2 mL of Ni–NTA Superflow resin (Qiagen) in an orbital shaker at 4 °C at 50 rpm for 60 min, and packed into a suitable chromatographic column. After passing 40 mL of wash buffer (60 mM Imidazole, 100 mM HEPES, 30 mM NaCl, pH 7.5), the recombinant proteins were recovered with elution buffer (300 mM Imidazole, 100 mM HEPES, 300 mM NaCl, pH 7.5). Eluted protein samples were concentrated and further purified by gel filtration (Superdex 200, 5 × 150 mm column) equilibrated with 25 mM HEPES, 250 mM NaCl, pH 6, with a solvent flow rate of 0.5 mL/min.

### Circular dichroism (CD)

Far-UV (190–250 nm) CD measurements were performed with a Jasco J-815 CD Spectrometer (JASCO Corporation, Japan) using a 1--mm path length quartz cell using protein samples at a final concentration of 2 μM in 10 mM of sodium phosphate buffer, pH 8.0. The spectra were recorded at a scan speed of 50 nm/min and at time response of 1 s. An average of 9 spectra were recorded, the buffer baseline was subtracted and after Savitzky–Golay smoothing using the CDTools software [32], the processed spectra were deconvoluted by the ContinII software [33] with database 4 [34] available in the DichroWeb analysis server [35]. The normalized root mean-square deviation (NRMSD) goodness-of-fit parameter was < 0.15, suggesting that the calculated spectra are a good approximation of the experimental data [36].

### Enzymatic assays and determination of the kinetic parameters

The hydrolysis of carboxymethylcellulose (CMC, Sigma-Aldrich Chem. Co.) and barley β-glucan [28] was assayed at 50 °C in Bis–Tris buffer, pH 6.0, in a final volume of 0.6 mL. The reducing sugars released were quantified using the dinitrosalicylic acid (DNS) method [37]. Controls with heat-inactivated enzyme were included in all enzymatic assays. The experimental conditions (reaction times, enzymatic units) were adjusted to guarantee the estimation of initial velocities. One enzyme unit (U) was defined as the amount of enzyme that releases 1 μmol of product per min. The specific activity was defined as the enzyme units per milligram of total protein (U mg^−1^). All enzymatic assays were performed in duplicate. Maximum velocities (*V*_max_) and apparent affinity constants (*K*_0.5_) for the hydrolysis of CMC and β-glucan by BsCel5A-RtCBM11 and BsCel5A-CBM3 were estimated using the SigrafW Software [38]. All experimental kinetic curves were repeated three times using different recombinant enzyme preparations, where each experimental point was assayed in duplicate. The kinetic parameters presented are calculated values and are given as the mean ± SD of the three replicates (*n* = 3).

### Site-directed spin labeling and EPR measures

The purified proteins were transferred to labeling buffer (40 mM Hepes, 300 mM NaCl, pH 8.0), and immediately incubated with a 5-fold molar excess of the spin-labeled 2,5-dihydro-2,2,5,5-tetramethyl-3-[[(methylsulfonyl)thio]methyl]-1H-pyrrol-1-yloxy (MTSSL) for 24 h at 25 °C. The modified protein was separated from excess free spin label with a Superdex 200 column (5 × 150 mm) eluted with 40 mM HEPES, 300 mM NaCl, and pH 8.0 buffer. The spin-labeled proteins were concentrated to 200 μM by ultrafiltration.

Continuous wave EPR spectroscopy was performed at room temperature using a JEOL JES-FA200 equipped with temperature control and a cylindrical cavity operating in X band mode. Spectral analyses were performed according to empirical parameters as previously described (Columbus and Hubbell, 2004). The Ms mobility parameter was calculated as function of the central line width as *M*s = (*δ*_exp_^−1^ − *δ*_i_^−1^)/(*δ*_m_^−1^ − *δ*_i_^−1^), where *δ*_exp_ is the value of the central line width of R1 in the MTSSL-labeled protein. The *δ*_i_ = 8.4 G and *δ*_m_ = 2.1 G are the values of the greatest degree of either immobilization or mobility of sites observed in a protein, respectively, as obtained in studies with rhodopsin [14, 18].

Solutions containing labeled proteins free in solution, and in the presence of either purified β-glucan or pre-treated sugarcane cell wall samples were prepared and drawn into capillary tubes. The final protein concentration was 100 μM, and the β-glucan concentration was varied from 0.1 to 5 mg/mL. The sugarcane cell wall preparations were used at a concentration of 1 mg/mL. The titration curve was fitted to a sigmoidal Hill function by non-linear regression using the program Microcal Origin version 8.0, to yield estimates of the association constant (*Ka*).

### Sugarcane cell wall preparation, fractionation, and enzymatic assay

Five hundred milligrams of sugarcane bagasse culms were hammer-milled to a 0.5 mm particle size, and extracted 6 times with 25 mL of 80% (v/v) ethanol at 80 °C for 20 min to remove sugars and other soluble compounds. The alcohol insoluble residue (AIR) was washed with distilled water and dried at 60 °C for 24 h [39]. The AIR was subjected to two consecutives extractions with 25 mL of 0.5 M ammonium oxalate pH 7.0 at 80 °C for 3 h with continuous stirring. The solid oxalate-extracted residue was recovered by centrifugation at 13,000*g* for 30 min and dried at 60 °C for 24 h [39]. Subsequent extraction with DMSO followed a previously reported protocol [40], where approximately 4 g of oxalate-extracted sugarcane bagasse was subjected to two DMSO extractions using a ratio of 14 mL DMSO per gram of biomass at room temperature with continuous stirring at 20 rpm at 70 °C for 2 h. The solids fraction containing the DMSO-extracted biomass was recovered and washed with ethanol and dried [40]. Biomass delignification was performed as previously described [41] where approximately 10 g of milled sugarcane bagasse was suspended in 100 mL of water to which was added 4 g NaClO_2_, followed by the addition of 500 mL of glacial acetic acid. The mixture was incubated at 60 °C for 3 h. At hourly intervals, 4 g of NaClO_2_ was added until a total of 0.80 g of NaClO_2_/g of biomass was reached. The mixture was vacuum filtered and the solid fraction was washed with deionized water and dried at 50 °C for 24 h [41, 42].

The activity of the enzymes against AIR and all the treated lignocellulosic substrates was performed using a 1% w/v suspension of the treated substrate prepared in 50 mM Bis–Tris buffer, pH 6.0, buffer, 100 μM of either BsCel5A-CBM3 or BsCel5A-RtCBM11 at 50 °C for 24 h in an orbital shaker at 200 rpm. The release of total reducing sugars was measured by the DNS method [37].

## Results and discussion

### Exchanging the CBM3 domain of the BsCel5A for RtCBM11 increases catalytic efficiency

The cysteine mutants of the *Bscel5A*-*CBM3* and *Bscel5A*-*RtCBM11* nucleotide coding sequences encode polypeptides of 494 and 514 amino acids, respectively, and were successfully expressed as soluble proteins in *E. coli* Roseta (DE3). The molecular masses of the cysteine mutant proteins BsCel5A-CBM3 and BsCel5A-RtCBM11 were 54,787.9 and 56,897.3 Da, respectively, including the polyhistidine sequence added to the *N*-termini derived from the pET28a plasmid. The relative molecular mass of both enzymes estimated by gel filtration was approximately 55 kDa (data not shown), and it was therefore assumed that both enzymes are monomeric. The far-UV CD spectra of the pure enzymes show a maximum at approximately 195 nm, a minimum around 215 nm, and a shoulder at 225 nm (Additional file 1: Figure S1) that are characteristic of spectra from proteins with a mixed α-helix and β-strand secondary structure content. The secondary structure content (inset to Additional file 1: Figure S1) estimated by deconvolution of the CD spectra of the BsCel5A-CBM3 was 26.4% α-helix and 20.7% β-strand and for the BsCel5A-CBM3 was 26.6% α-helix and 22.3% β-strand. These values are consistent with the sum of the secondary structure assignments from the crystallographic structure of the BsCel5A catalytic domain (PDB 3PZV, [27]) with either the CBM3 (PDB 2L8A [27], sum of 22.5% α-helix and 21.5% β-strand) or the RtCBM11 (PDB 1V0A [43], sum of 21.2% α-helix and 27.0% β-strand).

**Fig. 1:**
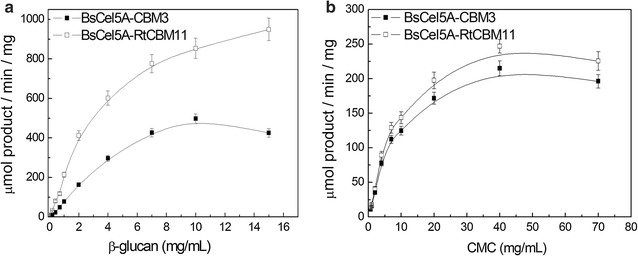
The catalytic activity of the cysteine mutants BsCel5A-CBM3 and BsCel5A-RtCBM11 against (**a**) barley β-glucan and (**b**) CMC

The endoglucanase activity with different concentrations of either β-glucan or CMC substrates (see Fig. 1) was higher against the β-glucan for both chimeric enzymes, confirming that the catalytic domain prefers the natural β-glucan as a substrate. Analysis of the kinetic parameters for CMC and β-glucan hydrolysis (see Table 1) reveals that the *V*_max_ and *k*_cat_ values for β-glucan hydrolysis were almost 2-fold higher for the BsCel5A-RtCBM11 as compared to the BsCel5A-CBM3 enzyme, and was accompanied by a slight decrease in the *K*_0.5_ (from 2.88 to 2.66 mg/mL) of the BsCel5A catalytic domain when the CBM3 was replaced by RtCBM11. The catalytic efficiency (*k*_cat_/*K*_0.5_) of β-glucan hydrolysis was 53% greater for BsCel5A-RtCBM11 as compared to BsCel5A-CBM3. Both enzymes presented similar *K*_0.5_ values for CMC hydrolysis, suggesting that the catalytic domain can maintain hydrolytic function independent of the associated CBM. This result is in agreement with the previously known affinities of the CBMs for the polysaccharide used in this study, where RtCBM11 shows a higher affinity for β-glucan as compared to CBM3, and neither CBM3 nor RtCBM11 presents significant binding to CMC [44].

**Table 1:** Kinetic parameters for β-glucan and CMC hydrolysis by cysteine mutants BsCel5A-CBM3 and BsCel5A-CBM11 enzymes

Enzyme	Substrate	*V*_max_ (U mg^−1^)	*K*_0.5_ (mg mL^−1^)	*K*_cat_ (s^−1^)	*K*_cat_/*K*_0.5_ (mL mg^−1^ s^−1^)
BsCel5A-CBM3	β-glucan	498.1 ± 20.1	2.88 ± 0.05	472.34 ± 20.1	164.0
	CMC	218.7 ± 10.4	6.97 ± 0.15	199.70 ± 10.4	28.65
BsCel5A-RtCBM11	β-glucan	985.7 ± 40.2	2.66 ± 0.04	934.72 ± 40.2	351.4
	CMC	251.5 ± 9.6	6.98 ± 0.22	238.49 ± 9.6	34.16

### Binding of the BsCel5A-RtCBM11_Y151R1_ to β-glucan is confirmed by EPR-SDSL

Experimental strategies used to study polysaccharide binding to CBMs include affinity electrophoresis, insoluble polysaccharide pulldown, and NMR [45]. Here we have introduced a novel and complementary method to investigate CBM binding dynamics based on spin labeling electron paramagnetic resonance (SDSL-EPR) experiments. We have produced single cysteine mutants in the carbohydrate-binding regions of the CBM domains in both the BsCel5A-CBM3 and BsCel5A-RtCBM11, and modified these cysteines with the spin probe MTSSL. The labeled proteins were used to analyze changes in the dynamics of both enzymes in the presence of the natural substrate β-glucan and several pre-treated biomass samples.

**Fig. 2:**
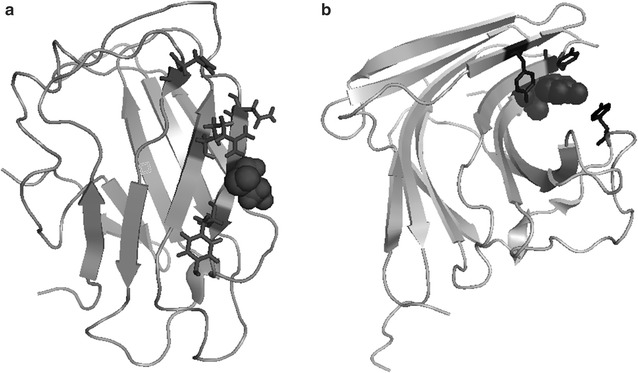
Ribbon representations of the structures of the single cysteine mutants of (**a**) BsCBM3 and (**b**) RtCBM11 created by SWISS-MODEL using the PDB file 2L8A and 1V0A, respectively, as templates. The residues modified by the MTSSL probes for EPR studies are depicted as dark gray spheres and the residues involved in carbohydrate binding are indicated by black sticks

Type A CBMs interact with the smooth surface of crystalline cellulose with a planar hydrophobic carbohydrate-binding site [10, 12], Type B CBMs bind within soluble extended polysaccharide chains (*endo*-binding) in a cleft on the protein structure, whereas Type C CBMs have shortened pockets that bind to the termini of the polysaccharides (*exo*-binding) [46]. The crystal structures of the Type A *Bacillus subtilis* CBM3 (BsCBM3) and the Type B *Ruminiclostridium thermocellum* CBM11 (RtCBM11) are presented in Fig. 2. The CBM3 domain from BsCel5A presents a β-sandwich fold, with a core formed by two β-sheets of four and five β-strands, and the flat surface typical of Type A CBMs comprises the four β-strands that includes the charged and polar residues that make contacts with the surface of aggregated cellulose [39]. In contrast, the RtCBM11 has a “jelly roll” architecture consisting of two six-stranded anti-parallel β-sheets, which form a curved surface that defines a single ligand-binding cleft that is typical for Type B CBMs [43].

The CBM3 domain from BsCel5A and the RtCBM11 structures presented in Fig. 2 also highlights the residues labeled with the MTSSL probe in the spin-labeling EPR experiments. The crystal structure of the wild-type CBM3 domain from BsCel5A (PDB code 2L8A) reveals two unpaired cysteine residues (C405 and C412). Residue C412 in the BsCel5A-CBM3 was mutated to alanine resulting in a protein containing the single cysteine C405. The wild-type RtCBM11 does not include any cysteine residues, and analysis of the crystal structure (PDB code 1V0A) reveals that the Y151 is located in the substrate binding cleft, which is convenient for reporting local changes by SDSL-EPR. This residue was mutated in the BsCel5A-RtCBM11 to generate the Y151C single cysteine mutant. Both single cysteine mutant proteins were successfully labeled with the MTSSL spin probe to generate the BsCel5A-RtCBM11_Y151R1_ and BsCel5A-CBM3_C405R1_ and used in SLDL-EPR experiments.

**Fig. 3:**
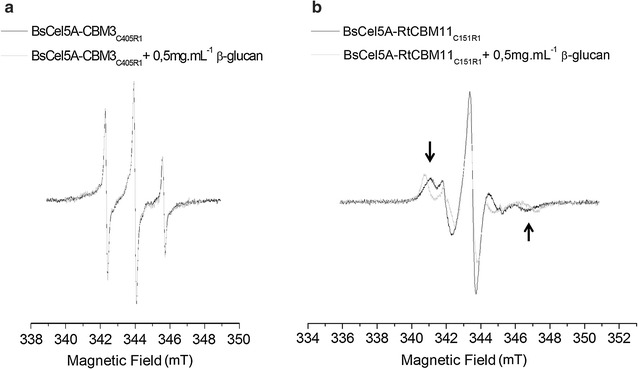
Measured EPR spectra of (**a**) BsCel5A-CBM3_C405R1_ and (**b**) BsCel5A-RtCBM11_Y151R1_ both free in solution and in the presence of β-glucan. The spectra are superimposed and normalized to the total area to emphasize the difference in line width and shape. The arrows highlight the increase in the out-peak separation of the spectrum due to β-glucan binding and immobilization of the spin-labeled side chain

An empirical analysis of the mobility of the MTSSL probes exploits the fact that the EPR spectra are doubly integrated, resulting in spectra that are normalized with respect to total area between samples with an equal number of spin labels [14]. In the case of nitroxide radicals such as MTSSL tumbling free in solution, the EPR spectrum is defined by three narrow well-resolved resonance lines. When the nitroxide radicals are covalently linked to cysteine side chains in a protein, the line shape of the EPR spectra is modified according to the dynamics of the region, where dynamically restricted labeled side chain show less intense and broadened lines [14, 18, 47]. Figure 3 presents the EPR spectra of BsCel5A-RtCBM11_Y151R1_ and BsCel5A-CBM3_C405R1_ in the presence and absence of β-glucan. All spectra show three prominent narrow lines typical of nitroxide spin probes (Fig. 3a) that are typical of highly mobile probes. The EPR spectra of BsCel5A-CBM3_C405R1_ are not significantly altered in the presence of β-glucan, demonstrating that the dynamics of the labeled residue is unchanged and suggesting the absence of binding of β-glucan by CBM3 domain. In contrast, the EPR spectrum of BsCel5A-RtCBM11_Y151R1_ in the absence of β-glucan is typical of a probe showing intermediate dynamics (Fig. 3b). In the presence of β-glucan, the EPR spectra show significant changes with a clear increase in the out-peak separation (arrows in Fig. 3b) suggesting a more immobilized nitroxide radical. Therefore, the spectral changes promoted by the presence of β-glucan indicate that the labeled residue experiences a restriction in mobility due to the interaction of the RtCBM11 domain with β-glucan.

### EPR-SDSL can measure the binding affinity of BsCel5A-RtCBM11_Y151R1_ to β-glucan

To estimate the degree of mobility changes of the labeled side chains, the value of the Ms parameter was calculated from the nitroxide EPR spectra as the inverse of the central line width [14, 18]. An increase of the Ms value is directly correlated with the increase in the mobility experienced by the labeled side chain. The Ms values for BsCel5A-CBM3_C405R1_ and BsCel5A-RtCBM11_Y151R1_ in the absence of β-glucan (see Table 2) are 1.22 and 0.49, respectively, which is consistent with the surface exposure and higher mobility of the spin label in the BsCel5A-CBM3_C405R1_.

**Table 2:** Ms parameter estimation from EPR spectra of labeled proteins

Labeled protein	**Ms parameter**
BsCel5A-CBM3_C405R1_ in solution	1.22
BsCel5A-RtCBM11_Y151R1_ in solution	0.49
BsCel5A-RtCBM11_Y151R1_ + milled sugarcane	0.49
BsCel5A-RtCBM11_Y151R1_ 1 + OA residual fraction	0.45
BsCel5A-RtCBM11_Y151R1_ 1 + AIR fraction	0.47
BsCel5A-RtCBM11_Y151R1_ 1 + DMSO residual fraction	0.39
BsCel5A-RtCBM11_Y151R1_ 1 + delignificated fraction	0.29

**Fig. 4:**
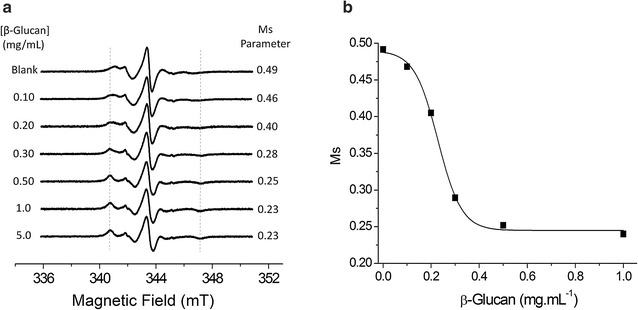
**a** Measured EPR spectra of the BsCel5A-RtCBM11_Y151R1_ in solution in the absence and in the presence of increasing concentrations of β-glucan. The dash lines indicate the spectral lines in BsCel5A-RtCBM11_Y151R1_ spectra that are indicative of the immobilized protein population. **b** The Ms parameter from spectra of the BsCel5A-CBM11_Y151R1_ as a function of β-glucan concentration. The light gray line shows the fitted curve which yields a *Ka* value of 0.22 mg/mL

The titration of BsCel5A-RtCBM11_Y151R1_ with β-glucan was accompanied by EPR spectroscopy (Fig. 4a). An increase in β-glucan concentration results in an increase of the out-peak separation with a corresponding broadening of the spectral line shape, as indicated by the decrease in Ms values (see Fig. 4b). The vertical lines in Fig. 4a are included as a guide to assist the accompaniment of the spectral features resulting from the immobilization of the spin probe on association of the protein with the β-glucan substrate. From the sigmoidal transition in Ms values, the β-glucan shows cooperative binding to the BsCel5A-RtCBM11_Y151R1_ (Fig. 4b), and reveals a saturating interaction at concentrations above 0.5 mg/mL of β-glucan with an association constant (*Ka*) of 0.23 ± 0.01 mg/mL. This value is consistent with the previously reported *Ka* value of 0.24 mg/mL for cooperative binding of the RtCBM11 protein to β-glucan as estimated from isothermal titration calorimetry experiments [43].

Comparison of the titration results from EPR with the results from reducing sugar release reveals that the RtCBM11 binding is saturated at β-glucan concentrations of less than 0.5 mg/mL, whereas the measured catalytic activity of the BsCel5A-RtCBM11 continues to increase even at a β-glucan concentration of 15 mg/mL. The high-affinity association of the Type B RtCBM11 within the β-glucan might be expected to facilitate the binding of the BsCel5-RtCBM11 and enhance catalysis by the active site domain. After hydrolysis, we propose that a reduced affinity of the RtCBM11 *exo*-binding at the polysaccharide terminus generated by hydrolysis favors dissociation even at high β-glucan concentrations, thereby liberating the enzyme for high-affinity *endo*-binding to the substrate. We note that no significant change was observed in the K_0.5_ between the BsCel5A-CBM3 and BsCel5A-RtCBM11 constructs, further indicating that the catalytic and carbohydrate-binding domains can function independently. It has previously been observed that a xyloglucan-specific endo-β-1,4-glucanase from *Aspergillus niveus* (XegA) fused to a xyloglucan-specific CBM44 domain from the CelJ of *R. thermocellum* (RtCBM44) exhibits a 30% increase in the catalytic efficiency for xyloglucan as compared to the parental enzyme [6]. It was proposed that the CBM in the XegA-RtCBM44 chimera alters the topology of the carbohydrate interaction in the active site of the XegA-RtCBM44 chimera thereby promoting a favorable conformation of the substrate in the catalytic domain [6].

**Fig. 5:**
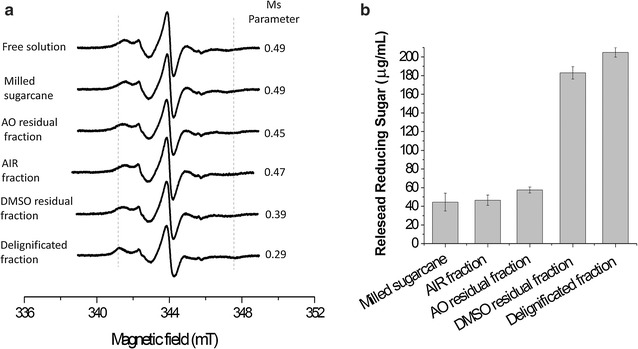
**a** Measured spectra of BsCel5A-RtCBM11_Y151R1_ in solution, and in the presence of different fractions of milled sugarcane bagasse or pre-treated fractions. The dash lines indicate the spectral lines in BsCel5A-RtCBM11_Y151R1_ spectra that are indicative of the immobilized protein population. **b** Total reducing sugar release after treatment of milled sugarcane bagasse or pre-treated fractions with the cysteine mutant BsCel5A-RtCBM11

The EPR technique is not only robust, but also has the advantage of selectively targeting defined paramagnetic centers [14] and is not subject to interference from scattering due to the presence of particulate material [15]. The use of EPR is therefore well suited to study the binding of spin-labeled proteins to lignocellulosic biomass. The lignocellulose composition of the sugarcane cell wall is 38–48% cellulose and β-glucan, 25–32% hemicelluloses (mainly xylan, xyloglucan, and arabinoxylan), and 17–24% lignin [48, 49]. The EPR spectrum of BsCel5A-RtCBM11_Y151R1_ in the presence of untreated milled sugarcane bagasse (Fig. 5) presents no significant changes as compared to the protein in solution, indicating that there is no interaction with this material. This is consistent with the intrinsic recalcitrance of untreated lignocellulose to enzymatic hydrolysis [50], which is a consequence of the high level of complexity of the plant cell wall [39] that restricts enzyme access to the constituent polysaccharides.

### Lignocellulose binding of BsCel5A-RtCBM11_Y151R1_ measured by EPR-SDSL correlates with reducing sugar release

The different components of the cell wall lignocellulose can be selectively extracted with alternative chemical treatments that break or rearrange the covalent and non-covalent linkages between polysaccharide structures [51]. These treatments reduce the polysaccharide crystallinity and increase the surface area, which facilitates enzyme access resulting in enhanced hydrolysis [52, 53]). Here we have performed a hierarchical cell wall deconstruction with chemical treatments that sequentially modifies the polysaccharide and lignin content of the biomass.

The first treatment is with ethanol and water to remove the soluble sugars and other soluble components to leave the alcohol insoluble residue (AIR). The EPR spectra of the BsCel5A-RtCBM11_Y151R1_ in the presence of this material (Fig. 5) did not present significant differences in the Ms parameter as compared to the free protein control in solution. The biomass was subsequently extracted with ammonium oxalate to release proteins and pectin (the AO residual fraction), and consequently the resulting material has a reduced content of neutral monosaccharides and uronic acids [39]). The EPR spectrum of the BsCel5A-RtCBM11_Y151R1_ collected with the AO residual fraction shows only slight modifications (Fig. 5). The next fractionating step was performed with DMSO to yield the DMSO residual fraction. In sugarcane cell walls, the xylan polymers contain β-1,4-linked d-xylose units that are modified as *O*-acetyl-4-*O*-methylglucurono-xylan, and the removal of this polysaccharide increases the efficiency of cellulose hydrolysis [54, 55]. The DMSO treatment removes the soluble cell wall xylan [40, 56]. The EPR spectrum of the BsCel5A-RtCBM11_Y151R1_ with the DMSO residual fraction shows a visible increase in out-peak separation and a reduced Ms value as a result of the interaction with RtCBM11 domain with the treated biomass (Fig. 5).

The final fractionation step is the removal of lignin, which has a supramolecular polymeric structure derived from aromatic groups linked by β-*O*-4a alkyl-ari-ether bonds [57, 58]. The role of lignin in the plant cell wall is both structural and to protect from microbial degradation [59], and it has been suggested that the removal of lignin is the key to the increased hydrolysis of lignocellulosic material [58, 60–63]. The EPR spectrum of the BsCel5A-RtCBM11_Y151R1_ in the presence of the delignificated cell wall fraction (Fig. 5) is visibly altered, showing a decrease in the Ms parameter due to reduced labeled side chain dynamics, and provides evidence for the interaction of the protein with the treated biomass.

These results were correlated with the results of total reducing sugar release using sugarcane bagasse after the same sequence of treatments as used in the EPR experiments. Reducing sugar release (Fig. 5b) on incubation of the BsCel5A-RtCBM11 with either AIR or with the AO residual fraction shows no significant difference when compared to the untreated milled bagasse. However, after the treatments for xylan and lignin removal from the bagasse, the total reducing sugar release increases by approximately 4.5- and 5-fold, respectively (Fig. 5b). The increased hydrolysis efficiency against both the DMSO residual fraction and the delignified sugarcane bagasse is strongly correlated with the increased BsCel5A-RtCBM11_Y151R1_ binding observed in the EPR experiments. It has been demonstrated that non-selective binding of hydrolytic enzymes to plant cell wall lignin can reduce their catalytic activity [8], and it has been suggested that this binding effect may present a major obstacle to the use of enzymes for the deconstruction of lignocellulose [64]. The majority of previous reports have estimated enzyme binding to biomass indirectly by measuring the residual catalytic activity in supernatants after incubation with biomass, whereas the present study has investigated the binding of BsCel5A-RtCBM11_Y151R1_ to treated sugarcane by direct measurement with EPR spectroscopy. In contrast to many previous reports, our results have demonstrated that only low levels of protein bind to samples in which lignin is present. Our results further indicate that in the case of the BsCel5A-RtCBM11, removal of the *O*-acetyl-4-*O*-methylglucurono-xylan with DMSO is the key treatment of the sugarcane resulting in both an increase in enzyme binding and a concomitant increase in reducing sugar release. The protective effect of the *O*-acetyl-4-*O*-methylglucurono-xylan suggests that the sequence of deconstruction of sugarcane cell wall requires the prior enzymatic or chemical removal of xylans for the exposure to hydrolysis by the β-glucanase. This implies that β-glucanase activity against cell wall lignocellulose would be enhanced in the presence of a xylanase, and indeed synergy between these two enzymes has been observed in the hydrolysis of wheat lignocellulose [65].

## Conclusions

Here we have shown that substitution of the naturally occurring CBM3 domain in the BsCel5-CBM3 by the RtCBM11 results in an increase in the hydrolytic efficiency of the enzyme. We have also shown for the first time through direct measurements by EPR spectroscopy that the CBM3 of the BsCel5A-CBM3 does not bind to β-glucan substrate, suggesting that the CBM3 and catalytic domains may act independently. The EPR measurements further reveal that the RtCBM11 of the BsCel5A-RtCBM11 displays high affinity for β-glucan, which is correlated with the increase in catalytic efficiency of the enzyme against this polysaccharide. To fully exploit the potential of EPR spectroscopy for the study of CBM binding in a biologically relevant environment, we have analyzed the association of the BsCel5A-RtCBM11 against a series of insoluble sugarcane bagasse fractions. The results suggest that hemicellulose impedes the binding of BsCel5A-RtCBM11 and the hydrolysis of the β-glucan in the sugarcane cell wall. This demonstrates the potential of EPR spectroscopy to study the interaction of hydrolases with complex insoluble lignocellulosic materials.

## Additional file

**Additional file 1: Figure S1.** Smoothed far ultraviolet circular dichroism spectra of the BsCel5A-CBM3 (black line) and BsCel5A-CBM11 (red line). The insert presents the percentage of secondary structure elements in both proteins as estimated by deconvolution using the ContinII software [33]. See the relevant section in “Materials and Methods” for further experimental details.

## References

[1] Kumar R, Singh S, Singh OV (2008). Bioconversion of lignocellulosic biomass: biochemical and molecular perspectives. J Ind Microbiol Biotechnol.

[2] Segato F, Damasio AR, Goncalves TA, Murakami MT, Squina FM, Polizeli M, Mort AJ, Prade RA (2012). Two structurally discrete GH7-cellobiohydrolases compete for the same cellulosic substrate fiber. Biotechnol Biofuels.

[3] Bhat MK, Bhat S (1997). Cellulose degrading enzymes and their potential industrial applications. Biotechnol Adv.

[4] Ogel ZB, Yarangumeli K, Du H, Ifrij I (2001). Submerged cultivation of scytalidium thermophilum on complex lignocellulosic biomass for endoglucanase production. Enzyme Microb Technol..

[5] Zhang YH, Lynd LR (2006). A functionally based model for hydrolysis of cellulose by fungal cellulase. Biotechnol Bioeng.

[6] Furtado GP, Santos CR, Cordeiro RL, Ribeiro LF, de Moraes LA, Damasio AR, Polizeli Mde L, Lourenzoni MR, Murakami MT, Ward RJ (2015). Enhanced xyloglucan-specific endo-beta-1,4-glucanase efficiency in an engineered CBM44-XegA chimera. Appl Microbiol Biotechnol.

[7] Kirk O, Borchert TV, Fuglsang CC (2002). Industrial enzyme applications. Curr Opin Biotechnol.

[8] Kaya F, Heitmann JA, Joyce TW (2000). Influence of lignin and its degradation products on enzymatic hydrolysis of xylan. J Biothecnol..

[9] Hashimoto H (2006). Recent structural studies of carbohydrate-binding modules. Cell Mol Life Sci.

[10] Boraston AB, Bolam DN, Gilbert HJ, Davies GJ (2004). Carbohydrate-binding modules: fine-tuning polysaccharide recognition. Biochem J.

[11] Ribeiro T, Ponte PI, Guerreiro CI, Santos HM, Falcao L, Freire JP, Ferreira LM, Prates JA, Fontes CM, Lordelo MM (2008). A family 11 carbohydrate-binding module (CBM) improves the efficacy of a recombinant cellulase used to supplement barley-based diets for broilers at lower dosage rates. Br Poult Sci.

[12] Varnai A, Siika-Aho M, Viikari L (2013). Carbohydrate-binding modules (CBMs) revisited: reduced amount of water counterbalances the need for CBMs. Biotechnol Biofuels.

[13] Guillen D, Sanchez S, Rodriguez-Sanoja R (2010). Carbohydrate-binding domains: multiplicity of biological roles. Appl Microbiol Biotechnol.

[14] Columbus L, Hubbell WL (2004). Mapping backbone dynamics in solution with site-directed spin labeling: GCN4-58 bZip free and bound to DNA. Biochemistry.

[15] Czogalla A, Pieciul A, Jezierski A, Sikorski AF (2007). Attaching a spin to a protein—site-directed spin labeling in structural biology. Acta Biochim Pol.

[16] Hubbell WL, McHaourab HS, Altenbach C, Lietzow MA (1996). Watching proteins move using site-directed spin labeling. Structure..

[17] Hubbell WL, Gross A, Langen R, Lietzow MA (1998). Recent advances in site-directed spin labeling of proteins. Curr Opin Struct Biol.

[18] Hubbell WL, Cafiso DS, Altenbach C (2000). Identifying conformational changes with site-directed spin labeling. Nat Struct Biol.

[19] Steinhoff H-J (2002). Methods for study of protein dynamics and protein-protein interaction in protein-ubiquitination by electron paramagnetic resonance spectroscopy. Front Biosci..

[20] Hubbell WL, Lopez CJ, Altenbach C, Yang Z (2013). Technological advances in site-directed spin labeling of proteins. Curr Opin Struct Biol.

[21] McHaourab HS, Lietzow MA, Hideg K, Hubbell WL (1996). Motion of spin-labeled side chains in T4 lysozyme. Correlation with protein structure and dynamics. Biochemistry.

[22] Borbat PP, Costa-Filho AJ, Earle KA, Moscicki JK, Freed JH (2001). Electron spin resonance in studies of membranes and proteins. Science..

[23] Couto SG, Cristina Nonato M, Costa-Filho AJ (2011). Site directed spin labeling studies of Escherichia coli dihydroorotate dehydrogenase *N*-terminal extension. Biochem Biophys Res Commun.

[24] Basso LGM, Mendes LFS, Costa-Filho AJ (2016). The two sides of a lipid-protein story. Biophys Rev..

[25] Steinhoff HJ (2004). Inter- and intra-molecular distances determined by EPR spectroscopy and site-directed spin labeling reveal protein–protein and protein–oligonucleotide interaction. Biol Chem.

[26] Altenbach C, Oh KJ, Trabanino RJ, Hideg K, Hubbell WL (2001). Estimation of inter-residue distances in spin labeled proteins at physiological temperatures: experimental strategies and practical limitations. Biochemistry.

[27] Santos CR, Paiva JH, Sforca ML, Neves JL, Navarro RZ, Cota J, Akao PK, Hoffmam ZB, Meza AN, Smetana JH (2012). Dissecting structure-function-stability relationships of a thermostable GH5-CBM3 cellulase from Bacillus subtilis 168. Biochem J.

[28] Temelli F (1997). Extraction and functional properties of barley B-glucan as affected by temperature an pH. Enginering..

[29] Yague E, Beguin P, Aubert JP (1990). Nucleotide sequence and deletion analysis of the cellulase-encoding gene celH of *Clostridium thermocellum*. Gene.

[30] Guerreiro CI, Ribeiro T, Ponte PI, Lordelo MM, Falcao L, Freire JP, Ferreira LM, Prates JA, Fontes CM (2008). Role of a family 11 carbohydrate-binding module in the function of a recombinant cellulase used to supplement a barley-based diet for broiler chickens. Br Poult Sci.

[31] Furtado GP. Structural determinants of the substrate specificity of CBMs (Carbohydrate Binding Modules) and their contributions to the action of glycosyl hydrolases. Ribeirao Preto; 2013.

[32] Lees JG, Smith BR, Wien F, Miles AJ, Wallace BA (2004). CDtool-an integrated software package for circular dichroism spectroscopic data processing, analysis, and archiving. Anal Biochem.

[33] Sreerama N, Woody RW (2000). Estimation of protein secondary structure from circular dichroism spectra: comparison of CONTIN, SELCON, and CDSSTR methods with an expanded reference set. Anal Biochem.

[34] van Stokkum IH, Spoelder HJ, Bloemendal M, van Grondelle R, Groen FC (1990). Estimation of protein secondary structure and error analysis from circular dichroism spectra. Anal Biochem.

[35] Whitmore L, Wallace BA (2004). DICHROWEB, an online server for protein secondary structure analyses from circular dichroism spectroscopic data. Nucleic Acids Res..

[36] Whitmore L, Wallace BA (2008). Protein secondary structure analyses from circular dichroism spectroscopy: methods and reference databases. Biopolymers.

[37] Miller GL (1959). Use of dinitrosalicylic acid reagent for determination of reducing sugar. Anal Biochem..

[38] Leone FA, Baranauskas JA, Furriel RP, Borin IA (2005). SigrafW: an easy-to-use program for fitting enzyme kinetic data. Biochem Mol Biol Educ.

[39] De Souza AP, Leite DCC, Pattathil S, Hahn MG, Buckeridge MS (2013). Composition and structure of sugarcane cell wall polysaccharides: implications for second-generation bioethanol production. Bioenerg Res..

[40] Rowley J, Decker SR, Michener W, Black S (2013). Efficient extraction of xylan from delignified corn stover using dimethyl sulfoxide. 3 Biotech..

[41] Saarnio J, Wathen K, Gustafsson C (1954). The structure of acidic xylan isolated from birch wood holocellulose. Acta Chem Scand.

[42] Naran R, Black S, Decker SR, Azadi P (2009). Extraction and characterization of native heteroxylans from delignified corn stover and aspen. Cellulose.

[43] Carvalho AL, Goyal A, Prates JA, Bolam DN, Gilbert HJ, Pires VM, Ferreira LM, Planas A, Romao MJ, Fontes CM (2004). The family 11 carbohydrate-binding module of *Clostridium thermocellum* Lic26A-Cel5E accommodates beta-1,4- and beta-1,3-1,4-mixed linked glucans at a single binding site. J Biol Chem..

[44] Foumani M, Vuong TV, MacCormick B, Master ER (2015). Enhanced polysaccharide binding and activity on linear beta-glucans through addition of carbohydrate-binding modules to either terminus of a glucooligosaccharide Oxidase. PLoS ONE.

[45] Cockburn D, Wilkens C, Dilokpimol A, Nakai H, Lewinska A, Abou Hachem M, Svensson B (2016). Using carbohydrate interaction assays to reveal novel binding sites in carbohydrate active enzymes. PLoS ONE.

[46] Gilbert HJ, Knox JP, Boraston AB (2013). Advances in understanding the molecular basis of plant cell wall polysaccharide recognition by carbohydrate-binding modules. Curr Opin Struct Biol.

[47] Dyszy F, Pinto AP, Araujo AP, Costa-Filho AJ (2013). Probing the interaction of brain fatty acid binding protein (B-FABP) with model membranes. PLoS ONE.

[48] Sanjuan R, Anzaldo J, Vargas J, Turrado J, Patt R (2011). Morphological and chemical composition of pith and fibers from Mexican sugarcane bagasse. Holz als Roh-und Werkstoff..

[49] Masarin F, Gurpilhares DB, Baffa DC, Barbosa MH, Carvalho W, Ferraz A, Milagres AM (2011). Chemical composition and enzymatic digestibility of sugarcane clones selected for varied lignin content. Biotechnol Biofuels.

[50] Himmel ME, Ding SY, Johnson DK, Adney WS, Nimlos MR, Brady JW, Foust TD (2007). Biomass recalcitrance: engineering plants and enzymes for biofuels production. Science..

[51] Amjed M, Jung HG, Donker JD (1992). Effect of alkaline hydrogen peroxide treatment on cell wall composition and digestion kinetics of sugarcane residues and wheat straw. J Anim Sci.

[52] Balat M, Balat H, Öz C (2008). Progress in bioethanol processing. Progr Energy Combust Sci..

[53] Jørgensen H, Kristensen JB, Felby C (2007). Enzymatic conversion of lignocellulose into fermentable sugars: challenges and opportunities. Biofuels Bioprod Bioref..

[54] Gao Y, Xu J, Zhang Y, Yu Q, Yuan Z, Liu Y (2013). Effects of different pretreatment methods on chemical composition of sugarcane bagasse and enzymatic hydrolysis. Biores Technol.

[55] Gao D, Uppugundla N, Chundawat SP, Yu X, Hermanson S, Gowda K, Brumm P, Mead D, Balan V, Dale BE (2011). Hemicellulases and auxiliary enzymes for improved conversion of lignocellulosic biomass to monosaccharides. Biotechnol Biofuels.

[56] Morais de Carvalho D, Martinez-Abad A, Evtuguin DV, Colodette JL, Lindstrom ME, Vilaplana F, Sevastyanova O (2017). Isolation and characterization of acetylated glucuronoarabinoxylan from sugarcane bagasse and straw. Carbohydr Polym..

[57] Vanholme R, Demedts B, Morreel K, Ralph J, Boerjan W (2010). Lignin biosynthesis and structure. Plant Physiol..

[58] Mäkelä MR, EL. B, JK. M, SE. B, Vries RP, Hildén K. Fungal ligninolytic enzymes and their applications. Microbiol Spectr 2016; 4(6). 10.1128/microbiolspec.FUNK-0017-2016 28087936

[59] Moura JC, Bonine CA, de Oliveira Fernandes Viana J, Dornelas MC, Mazzafera P (2010). Abiotic and biotic stresses and changes in the lignin content and composition in plants. J Integr Plant Biol..

[60] Zhao X, Song Y, Liu D (2011). Enzymatic hydrolysis and simultaneous saccharification and fermentation of alkali/peracetic acid-pretreated sugarcane bagasse for ethanol and 2,3-butanediol production. Enzyme Microb Technol..

[61] Sun Y, Cheng J (2002). Hydrolysis of lignocellulosic materials for ethanol production: a review. Biores Technol.

[62] Fonseca-Maldonado R, Ribeiro LF, Furtado JP, Arruda LM, Meleiro LP, Alponti SP, Botelho-Machado C, Vieira DS, Bonneil E, Furriel RPM (2014). Synergistic action of co-expressed xylanase/laccase mixtures against milled sugar cane bagasse. Process Biochem.

[63] Godin B, Nagle N, Sattler S, Agneessens R, Delcarte J, Wolfrum E (2016). Improved sugar yields from biomass sorghum feedstocks: comparing low-lignin mutants and pretreatment chemistries. Biotechnol Biofuels..

[64] Rahikainen JL, Evans JD, Mikander S, Kalliola A, Puranen T, Tamminen T, Marjamaa K, Kruus K (2013). Cellulase-lignin interactions-the role of carbohydrate-binding module and pH in non-productive binding. Enzyme Microb Technol..

[65] Mathlouthi N, Saulnier L, Quemener B, Larbier M (2002). Xylanase, beta-glucanase, and other side enzymatic activities have greater effects on the viscosity of several feedstuffs than xylanase and beta-glucanase used alone or in combination. J Agric Food Chem..

