# Supplementary figures and images for: Lignocellulose binding of a Cel5A-RtCBM11 chimera with enhanced β-glucanase activity monitored by electron paramagnetic resonance
Source: Biotechnol Biofuels. 2017 Nov 14;10:269. doi: 10.1186/s13068-017-0964-0 (PMC5686792; doi:10.1186/s13068-017-0964-0)

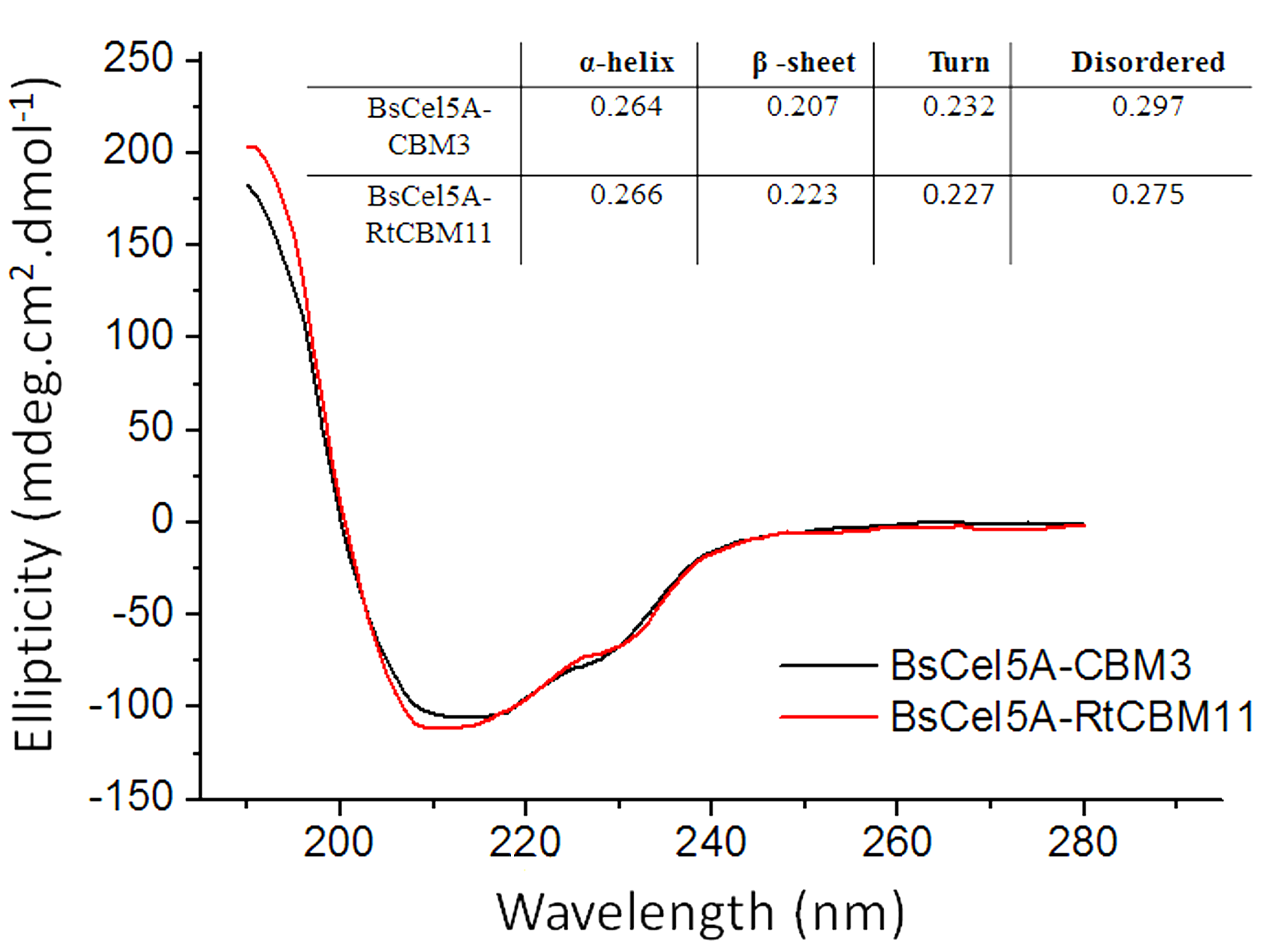

Supplement: Supplementary file 1 — Additional file 1: Figure S1. Smoothed far ultraviolet circular dichroism spectra of the BsCel5A-CBM3 (black line) and BsCel5A-CBM11 (red line). The insert presents the percentage of secondary structure elements in both proteins as estimated by deconvolution using the ContinII software [33]. See the relevant section in “Materials and Methods” for further experimental details. [file 13068_2017_964_MOESM1_ESM.tif]
